# Is Platelet-rich plasma superior to whole blood in the management of chronic tennis elbow: one year randomized clinical trial

**DOI:** 10.1186/2052-1847-6-12

**Published:** 2014-03-18

**Authors:** Seyed Ahmad Raeissadat, Seyed Mansoor Rayegani, Hossein Hassanabadi, Rosa Rahimi, Leyla Sedighipour, Khalil Rostami

**Affiliations:** 1 Department of Physical Medicine & Rehabilitation, Shahid Modarres Hospital, Shahid Beheshti University of Medical Sciences, Tehran, Iran; 2 Department of Physical Medicine & Rehabilitation, Mashhad University of Medical Sciences, Tehran, Iran; 3 Department of Plastic surgery, Shahid Moddares Hospital Shahid Beheshti University of Medical Sciences, Tehran, Iran

**Keywords:** Lateral humeral epicondylitis, Tennis elbow, Platelet rich plasma, Autologous whole blood

## Abstract

**Background:**

Lateral humeral epicondylitis, or ‘tennis elbow’, is a common condition with a variety of treatment options. Platelet-rich plasma (PRP) and Autologous Whole Blood (AWB) represent new therapeutic options for chronic tendinopathies including tennis elbow. The aim of the present study was to compare the long term effects of PRP versus autologous whole blood local injection in patients with chronic tennis elbow.

**Methods:**

Seventy six patients with chronic lateral humeral epicondylitis with duration of symptoms more than 3 months were included in this study and randomized into 2 groups. Group 1 was treated with a single injection of 2 mL of autologous leukocyte rich PRP (4.8 times of plasma) and group 2 with 2 mL of AWB. Tennis elbow strap, stretching and strengthening exercises were administered for both groups. Pain and functional improvements were assessed using visual analogue scale (VAS), Mayo score (modified Mayo Clinic performance index for the elbow) and pressure pain threshold (PPT) at 0, 4, 8 weeks and 6 and 12 months.

**Results:**

All pain variables including VAS, PPT and Mayo scores improved significantly in both groups at each follow up intervals compared to baseline. No statistically significant difference was noted between groups regarding pain, functional scores and treatment success rates in all follow up examinations (P >0/05).

**Conclusion:**

PRP and autologous whole blood injections are both effective methods to treat chronic lateral epicondylitis and their efficacy persisted during long term follow up. PRP was not superior to AWB in long term follow up.

## Background

Lateral elbow epicondylar tendinosis or tennis elbow is a common condition occurring at the common extensor tendon that originates from the lateral epicondyle in patients whose activities require strong gripping or repetitive wrist movements [1,2]. It causes pain and functional impairment in daily activities [1-3].

Histologic findings in chronic cases confirm that tendinosis is not an acute inflammatory condition but rather a failure of the normal tendon repair mechanism associated with angiofibroblastic degeneration [1-3]. Current research has produced several biological hypotheses regarding the cause of tendinosis based on histopathological, biochemical, and clinical findings that show cell apoptosis, angiofibroblastic features, or abnormal biochemical adaptations, largely suggesting that a failed healing response underlies the condition [4].

There are a variety of treatment options for this common condition [5,6]. The treatment is initially conservative. Numerous methods have been advocated to treat tennis elbow, including rest, anti inflammatory medications, bracing, physical therapy, ionotophoresis, extra corporal shockwave and botulinum toxin. Injections of corticosteroids, dry needling and various surgical techniques can been incorporated in refractory cases [6,7]. However, these traditional therapies do not alter the tendon’s poor healing properties secondary to poor vascularization [6]. Given the inherent nature of the tendon, new treatment options including Platelets Rich Plasma (PRP), autologous blood, prolotherapy, and extracorporeal shockwave therapy are aimed at inducing inflammation rather than suppressing it [1,7,8].

Platelet rich plasma is defined as a volume of the plasma fraction of autologous blood having a platelet concentration above baseline. Studies have shown that clinical efficacy can be expected with a minimum increase of 4 *times* this baseline [9].

Both PRP and autologous blood contain platelets with strong growth factors that may help in the healing process of chronic injuries. Known platelet growth factors stimulate the healing process and lead to partial modification of the damaged tissue [9-11]. The net results of PRP therapy in chronic tendinopathies are varied and hypothesized to include angiogenesis, increase in growth factor expression and cell proliferation, increase the recruitment of repair cells also, tensile strength [9,10]. Due to higher concentration of platelets in PRP than whole blood, it was suggested in some studies to have greater effect in the healing and repair process [12,13]. Various results have been published about applications of PRP in different fields such as skin and hair, ENT, orthopaedics etc. [14,15]. PRP use has also been evaluated in musculoskeletal disorders such as muscular injuries, achille and lateral epicondyle tendinopathies and with satisfactory results [8,12].

Some studies have shown that local injection of autologous whole blood has greater therapeutic effect than steroid injection in treating tennis elbow [10,11], also there are studies showing the greater efficacy of local autologous PRP than corticosteroids in relieving the symptoms of this disorder [9,12]. However, only a few studies have been conducted to compare the efficacy of these two treatments. In a comparative study of these 2 treatments conducted by.

Considering the high cost of autologous PRP therapy and lack of a long term study comparing autologous whole blood versus PRP injection, the present study was aimed to evaluate the long term efficacy of autologous whole blood injection as a less costly treatment versus PRP in patients suffering from chronic lateral epicondylitis.

## Methods

### Patients and setting

In this clinical trial, patients with signs and symptoms of chronic lateral epicondylitis during Sep 2011-Oct 2013 referring to our unit, were evaluated to enter this randomized, single blind study.

### Inclusion criteria

Criteria for inclusion in the study were chronic clinically diagnosed lateral epicondylitis (based on symptoms, site of tenderness and pain elicited with resisted active extension of the wrist in pronation and elbow extension); with duration of symptoms more than 3 months and pain severity with minimum score of 5 (based on 10 scale VAS (Visual Analogue Score) [16].

### Exclusion criteria

Patients older than 70 years old, any recent febrile or infectious disease, history of any malignancy (including hematologic and non hematologic malignancies), carpal tunnel syndrome, other peripheral nerve injury such as radial nerve injury, cervical radiculopathy, systemic illnesses including ischemic heart disease, diabetes, rheumatoid arthritis, hepatitis, any bony malformations, bony or articular lesions at elbow (diagnosed by radiographic imaging), history of autoimmune and platelet disorders, treatment with anticoagulant and anti-platelet medications 10 days before injection, consistent use of NSAIDs within 48 hours before procedure, use of systemic steroids during past 3 weeks, haemoglobin measures of less than 10 g/dl and platelet counts of less than 150,000 per micro liter, history of vasovagal shock, pregnancy or breastfeeding.

### Ethical considerations

From the ethical point of view, we gave written consent to all patients for inclusion in the study. The process of the treatment was simplified and explained to the patients, once the physician assured that the patient completely understood the study protocol and became aware of his rights during the study, the written consent form was signed or fingerprinted by the patient. The institutional review board of Shahid Beheshti University of Medical Sciences approved the protocol of this study. The process of treatment had no harm for their health, and they had authority to stop the process of treatment.

In case of very rare incidence of side effects associated with PRP or autologous blood injection (persistent pain and swelling, infection and fibrosis or any neuromuscular complications at injection site) patients had access to the project’s physician in order to contact him if they encountered any of the possible adverse reactions to injection.

### Randomization and patients’ enrolment

The block covariate adaptive randomization method is designed to randomize subjects into the treatment groups. This led to equal sample sizes within each group and balance of the important covariates. Thus, a new participant is sequentially assigned to particular treatment groups by taking into account the specific matched covariates and previous assignments of participants.

### Intervention

#### Group 1 (Autologous PRP group)

The treatment protocol for patients in this group was a single injection of 2 mL of autologous PRP, deep at the origin of wrist extensors, into maximal tenderness point at elbow region under aseptic technique.

### PRP preparation

For the process of PRP preparation and injection, participants were referred to Shahid Modarres hospital laboratory. The PRP processing was done using the Rooyagen kit (made by Arya Mabna Tashkhis Corporation, RN: 312569). For preparing 2 ml of PRP with concentration of 4-6 times the average normal values, 20 ml of blood was first collected from the patient’s upper limb cubital vein using an 18 G needle. Then 2 ml of ACD-A was added to the sample as an anticoagulant. One ml of the blood sample was sent for complete blood count. The rest of the sample passed two stages of centrifuge (first with 1600 rpm for 15 minutes for separation of erythrocytes and next with 2800 rpm for 7 minutes in order to concentrate platelets). The final product was 2 ml of PRP containing leukocytes (leukocyte rich PRP). The PRP quantification and qualification procedure was performed using laboratory analyzer Sysmex KX 21 and swirling and if approved, the injection was performed [17]. We did not use exogenous factor for the process of platelets activation.

### PRP injection

The patient is placed in an appropriate and comfortable position that allows for sterility and access to the site of injection.

The skin of the injection site was prepped and draped and the liquid PRP was injected in a sterile condition using a 18 G needle. The patient received a PRP injection at maximal point at elbow using a peppering technique spreading in a clock-like manner to achieve a more expansive zone of delivery.

#### Group 2 (Autologous whole blood)

Group 2 treatment protocol included a single injection of 2 mL of autologous peripheral whole blood under the same technique as the PRP group.

Two ml of lidocaine 1% was injected 8 minutes before PRP or whole blood injection for patients in both groups.

No cortisone or nonsteroidal anti-inflammatories were prescribed during follow-up. For pain relief only, oral paracetamol and ice therapy were used. Patients of both groups were requested to refrain from heavy labor activities for a week. Tennis elbow strap (Oppo trademark) was administered for all patients and they were instructed to apply the strap 2 centimetres below the maximal tenderness point at elbow.

The patients were followed via weekly telephone calls and instructed how to use elbow splint and perform exercises. Three days after the injection, each patient was asked to start a simple program of extensor muscles stretching and 2 weeks after injection eccentric loading exercises were prescribed to be performed on an individual basis twice every day for 5 weeks. The patients were allowed to perform full activities of daily living after 4 weeks.

### Outcome measures

#### Pain intensity

Pain severity was evaluated before injection and re-evaluation was done at 4, 8 weeks, 6 and 12 months after the injection. Visual analogue pain scale (VAS) (range, 0 [no pain] to 10 [agonizing pain]). The validity and reliability of self-rating scales like the VAS have previously been well described [16,18]. Modified Mayo Clinic performance index score was used to evaluate functional outcome after the treatment.

### Functional outcome measures

#### Modified Mayo clinic performance index

“Modified Mayo Clinic performance index” for the elbow was used as a valid and reliable measure to evaluate the functional improvement after therapy [19,20]. The Mayo Clinic performance index for the elbow has 4 parameters: Pain, motion, stability and daily function. The maximum score is 100 and the minimum index is 0, the results are interpreted as excellent (> = 90), good (75-89), fair (60-74) and poor (<60). The pain parameter carries the highest points (45) [19]. The modified mayo questionnaire was very specific to changes in elbow function. The questions were found to be reliable, reproducible and sensitive to change in elbow function [18]. Its construct validity is good for patient-rated variables and excellent for physician-rated variables. A minimal clinically important difference of 15 was reported for patients with rheumatoid arthritis after arthroplasty or synovectomy [20]. Mayo questionnaire was filled out via interviewing each patient at each follow up evaluations.

#### PPT

Pressure Pain Threshold (PPT) was assessed by an algometer, Commander trademark. The PPT test is precise and reliable measurement for assessing pain (Cronbach’s alpha ≥ 0.92). Pressure algometry has been shown to have good validity when assessed by pain and disability questionnaires [21]. The algometer is comprised of a gauge attached to a hard rubber tip. Pressure was applied though the rubber surface area of 1 cm^2^ at a rate of 2 Kg/Cm^2^ per second. The instrument was placed perpendicular to the skin’s surface. In each algometric assessment, we tested PPT at two different sites with 2 centimetres distance from each other at lateral epicondyle (site of maximal tenderness) and the mean of two values was considered as pain threshold. The method was demonstrated one time at each site before testing to ensure that the participants were familiar with the test. The participants were asked to indicate when the pressure became painful based on this definition: “When you feel the sensation changes from pressure to the slightest pain inform us”. Each measure site was tested three times with 2 minutes between each test. The scale unit was Kg/cm^2^.

### Statistical analysis

SPSS-16 (SPSS Inc Chicago, Illinois, United States of America) was used for data analysis. According to the Shapiro-Wilks normality tests, all variables had normal distribution so parametric tests including T-test, also Fisher’s exact, GLM: repeated measure and Greenhouse-Geisser tests were run to compare these variables between two groups. P-value less than 0.05 was considered significant. The assessors filling out the questionnaire and performing PPT, also the statistician were blinded to the group of the patient. The power of the study was considered 0.9 for determination of sample size.

## Results

**Figure 1:**
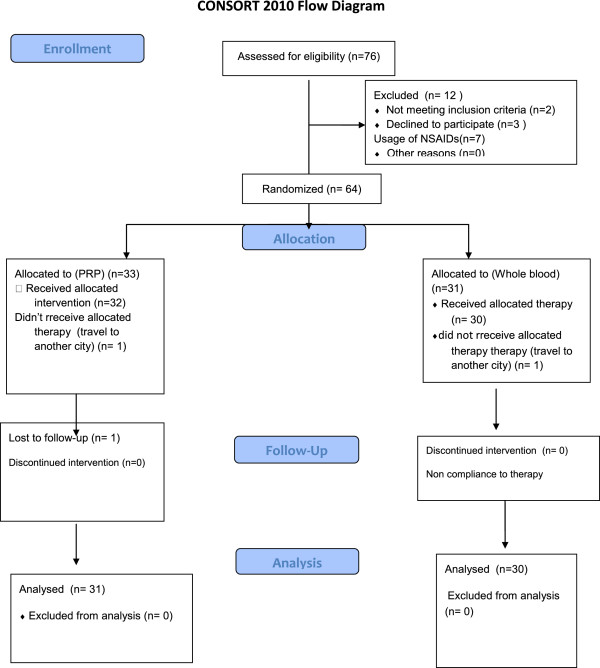
Consort flow diagram.

Seventy six patients were first evaluated for this study, but finally 61 patients completed the study and their data was analysed (thirty one patients in PRP and thirty in autologous group) (CONSORT flow chart Figure 1).

The mean age of patients was 45.3+/-5.9 years old. Forty seven patients were female (77%) and 14 patients were male (23%). All patients were right handed. The mean duration of symptoms in both groups was 14.5 ± 3 months. The patients’ characteristics at study entry were shown in Table 1. There were no between-group differences in demographic characteristics and pain intensity at baseline (Table 1).

**Table 1:** Demographic characteristics of patients in PRP and AWB groups

**Groups**	**(PRP)**	**(AWB)**	**P-values**	
Sex				
Male	8(26%)	6(20%)	P = 0.8	
Female	23(74%)	24(80%)	Fisher exact test	
Side of involvement				
Right	19(61)	22(73%)	P = 4	
Side	12(39%)	18(27%)	Fisher exact test	
Age	43 ± 6	44 ± 7	P = 0.4	
			T test	

### PRP characteristics

The mean platelets count of all patients at baseline was 250000 ± 53000/μl, which increased to 1227000 ± 250000/μl (4.8 times concentration) in PRP preparation. Leukocyte count was 6740 ± 1396/μl in PRP group and 6453 ± 1193/μl in AWB groups.

### Outcome measures

All outcomes including VAS and Mayo scores and PPT were measured before intervention. There were no between-group differences at baseline in pain intensity according to *VAS, Mayo and PPT scores. These scores* were measured at 4 and 8 weeks, also 6 and 12 months after initiating therapy in each group*.*

### VAS score

**Figure 2:**
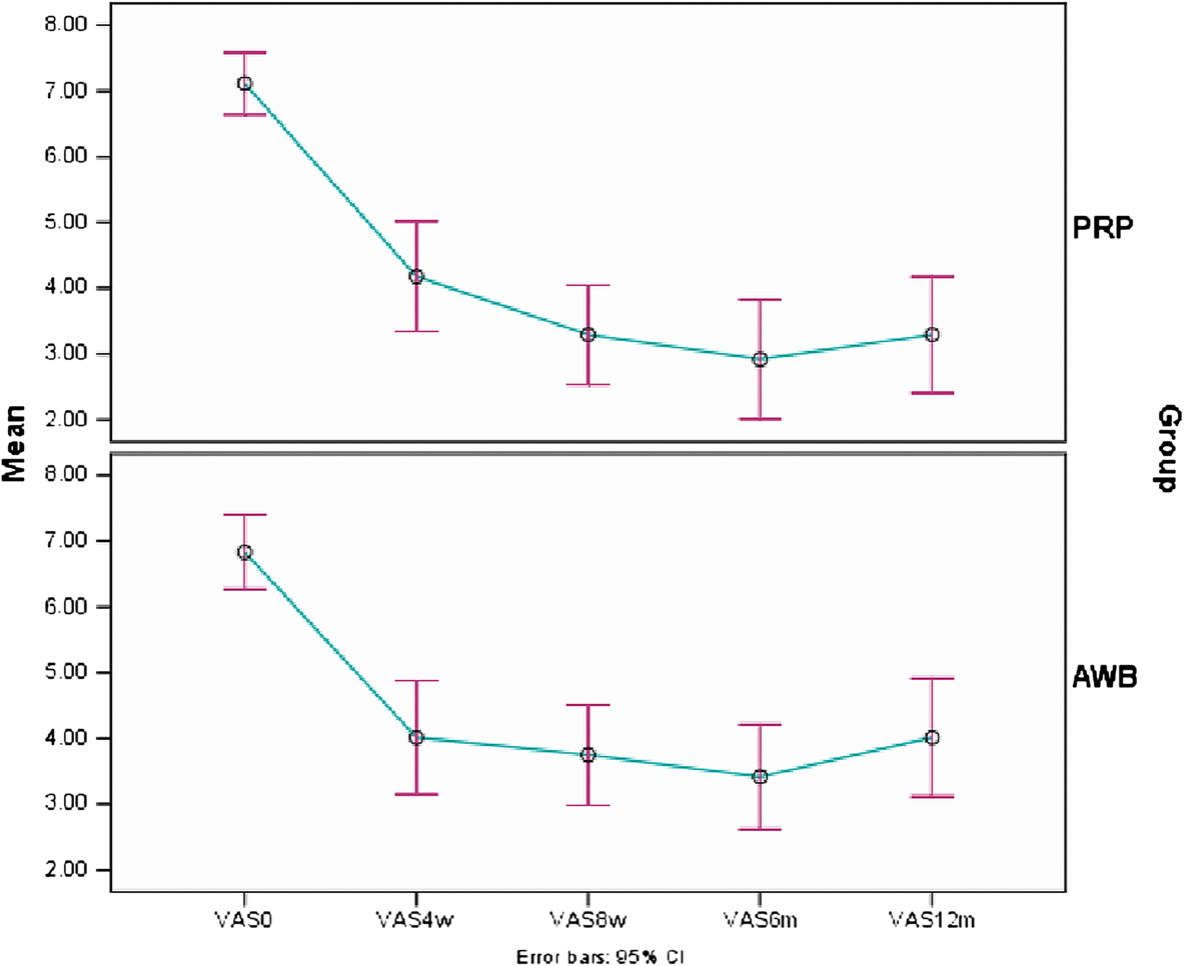
Mean (±sd) of VAS score in PRP and Autologous Whole Blood (AWB) groups at baseline, 4, 8 weeks, 6 and 12 months after therapy.

Mean VAS score decreased significantly in both PRP and AWB groups at each follow up evaluations and at 12 months after therapy compared to baseline. (P < 0.001) (Table 2, Figure 2).

**Table 2:** Mean of VAS score ± sd compared between PRP and AWB groups at baseline, 4, 8 weeks, 6 and 12 months after therapy

**Group**	**VAS baseline**	**VAS 4 w**	**VAS 8 w**	**VAS 6 m**	**VAS 12 m**
	**Mean ± sd**	**Mean ± sd**	**Mean ± sd**	**Mean ± sd**	**Mean ± sd**
PRP	7.1 ± 1.2	4.17 ± 2.2	3.29 ± 2.05	2.91 ± 2.47	3.29 ± 2.41
AWB	6.8 ± 1.5	4.01 ± 2.3	3.75 ± 2.05	3.41 ± 2.13	3.94 ± 2.42
P-Value	0.4	0.67	0.81	0.318	0.662

### Mayo score

#### Post intervention (12 month follow up)

**Figure 3:**
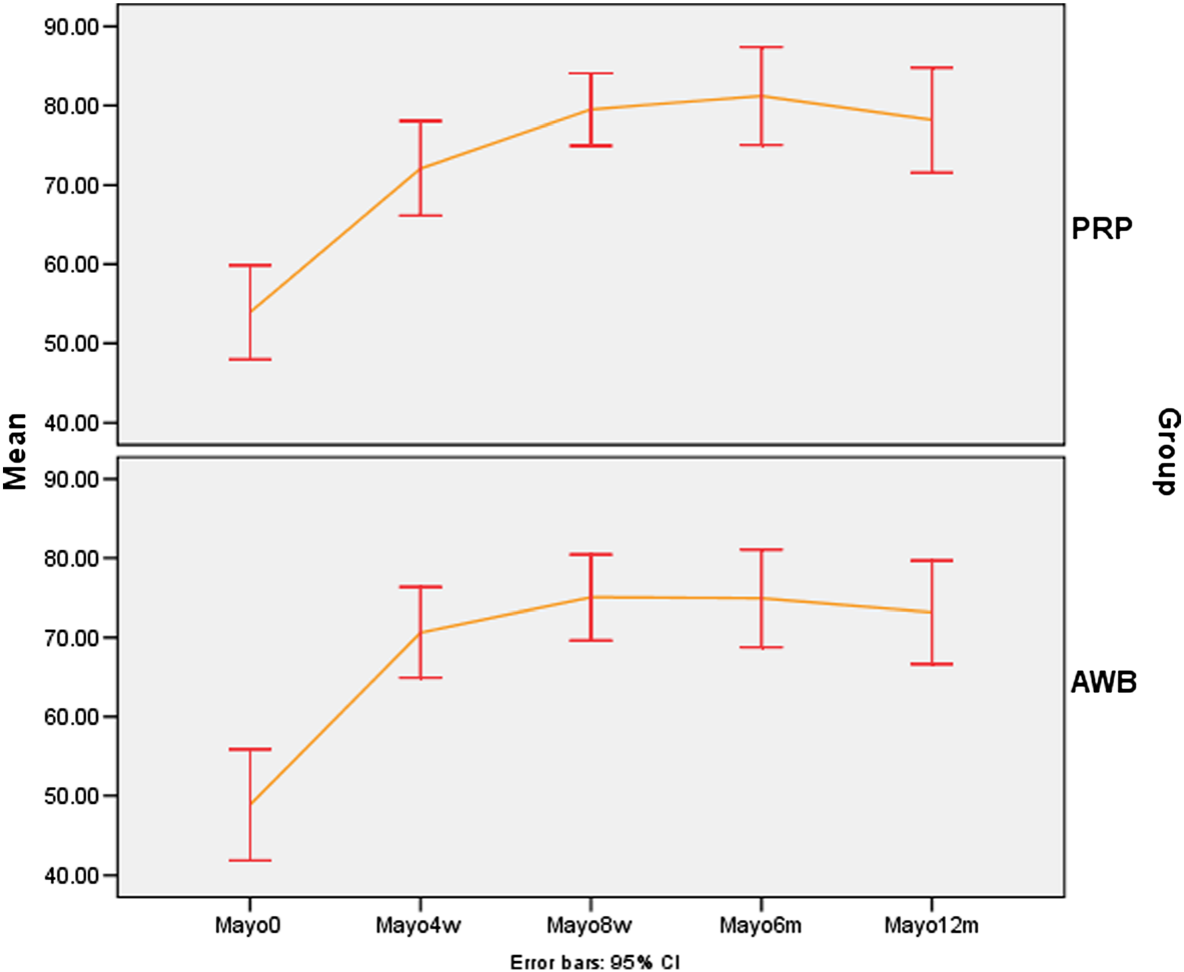
Mean (±sd) of Mayo score in PRP and Autologous Whole Blood (AWB) groups at baseline, 4, 8 weeks, 6 and 12 months after therapy.

Mayo score improved significantly in both PRP and AWB groups at each follow up evaluations and at 12 months after therapy compared to baseline (P < 0.001) (Table 3, Figure 3).

**Table 3:** Mean of Mayo score ± sd compared between PRP and AWB groups at baseline, 4, 8 weeks, 6 and 12 months after therapy

**Group**	**Mayo baseline**	**Mayo 4 w**	**Mayo 8 w**	**Mayo 6 m**	**Mayo 12 m**
	**Mean ± sd**	**Mean ± sd**	**Mean ± sd**	**Mean ± sd**	**Mean ± sd**
PRP	53.9 ± 16	72.09 ± 16	79.51 ± 12	81.20 ± 16	78.18 ± 18
AWB	48.8 ± 18	70.62 ± 15	75.04 ± 14	74.91 ± 16	73.16 ± 18
P-Value	0.3	0.59	0.597	0.59	0.59

### PPT score

#### Post intervention (12 month follow up)

**Figure 4:**
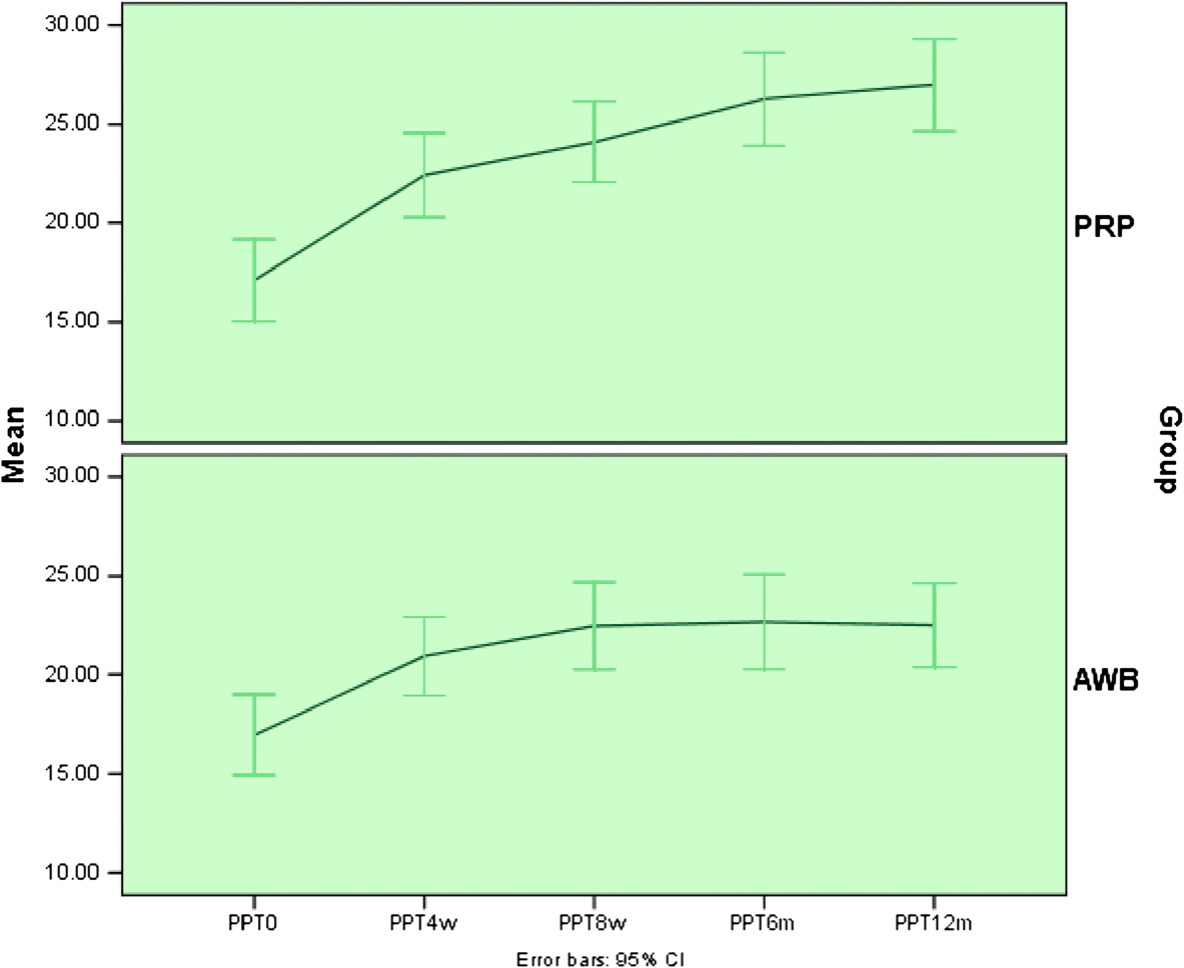
Mean (±sd) of PPT score in PRP and Autologous Whole Blood (AWB) groups at baseline, 4, 8 weeks, 6 and 12 months after therapy.

Mean PPT score improved significantly in both groups at each follow up evaluations and at 12 months after therapy compared to baseline (P < 0.002) (Table 4, Figure 4).

**Table 4:** Mean of PPT score ± sd compared between PRP and AWB groups at baseline, 4, 8 weeks, 6 and 12 months after therapy

**Group**	**PPT baseline**	**PPT 4 w**	**PPT 8 w**	**PPT 6 m**	**PPT 12 m**
	**Mean ± sd**	**Mean ± sd**	**Mean ± sd**	**Mean ± sd**	**Mean ± sd**
PRP	17 ± 5.6	22.4 ± 5.8	24 ± 5.5	26.2 ± 6.3	26.9 ± 6.3
AWB	16.9 ± 5.4	20.9 ± 5.3	22.4 ± 5.9	22.6 ± 6.4	22.5 ± 5.7
P-Value	0.9	0.207	0.207	0.207	0.207

#### Success rate

Success rate defined as 25% decrease in VAS score compared to baseline was achieved in both treatment groups in all 3 follow ups (Table 5).

**Table 5:** Success rate in two groups at 4 follow ups

**Groups**	**4 w**	**8 w**	**6 m**	**12 m**
PRP	19 (61%)	23 (75%)	25 (80.6%)	23 (75%)
AWB	19 (63%)	22 (73.3%)	23 (76.6%)	18 (60%)

### Between group comparisons

No statistically significant difference was noted between two groups regarding pain scores (VAS, Mayo and PPT) and success rate in all follow up examinations including 4,8 weeks and 6 and 12 months after initiating therapy (Tables 2, 3, 4 and 5, Figures 2, 3 and 4).

## Discussion

In the present study, PRP and AWB both lead to significant improvement in pain, function and pain pressure threshold in patients with chronic tennis elbow. However, this improvement was similar in both treatment groups which meant the effect of PRP therapy in tennis elbow management was shown to be almost same as AWB. Mayo score improvement reached minimal clinically important difference reported for Mayo score change following therapy in inflammatory joint disease in both treatment groups [20]. Also, success rate defined as 25% decrease in pain scores compared to baseline was achieved in both groups.

The efficacy of PRP injection for short term and long term pain relief in lateral epicondylitis was evaluated in previous studies [9,11,12].

There are many studies in favour of PRP in chronic tendinopathies. In 2006, Mirsha and his colleagues evaluated treatment of chronic severe elbow tendinosis with PRP. Eight weeks after the treatment, patients who had received PRP noted significant improvement in pain scores compared to control group [9]. The effectiveness of PRP compared with corticosteroid injections in patients with chronic lateral epicondylitis was also determined in a study by Peerbooms. He found that regarding pain reduction and functional improvement, corticosteroid was better initially and then declined, whereas the PRP group progressively improved, however this study also lacked a control group [12].

In 2013 Ahmad Z et al. carried out a systematic review of the current evidence on the effects of PRP in lateral epicondylitis on clinical outcomes. In this review, five randomized controlled trials were included. The largest randomized controlled trial found that PRP had significant benefit compared with corticosteroids with regard to pain and disabilities of the arm, shoulder and hand scores at 1 and 2 year time points. The review highlights the limited but evolving evidence for the use of PRP in lateral epicondylitis; however, further research is suggested by that study to understand the concentration and preparation that facilitates the best clinical outcome [22]. In another systematic review by Taylor DW seven studies were evaluated. This review demonstrated favourable outcomes in tendinopathies in terms of improved pain and functional scores. The authors concluded that PRP use in tendon and ligament injuries has several potential advantages, including faster recovery and possibly, a reduction in recurrence, with no adverse reactions described [23].

Contrary to the results of our study and the studies mentioned above, there are some studies showing no significant improvement in pain scores after PRP injection. Such a study was conducted by Shiple BJ conducted to compare the effectiveness of a single injection of platelet-rich plasma (PRP), glucocorticoid (GC), or saline in reducing pain in lateral epicondylitis. The pain intensity scale of the Patient-Rated Tennis Elbow Evaluation (PRTEE) questionnaire was the main outcome measure (least to most pain = 0-50 points) [24].

Krogh and his colleagues in 2013 randomized 60 subjects into three groups: PRP, corticosteroid or saline injection. All participants had had tennis elbow for at least 3 months. They found at three months no significant difference in terms of pain or functional improvement between the groups. The lack of sufficient number of platelets in PRP derivatives in above mentioned studies or different methods of PRP preparation might be one reason for not getting positive effects from PRP injection [25].

On the other hand, the efficacy of autologous whole blood injection in treatment of chronic tennis elbow has been evaluated in a number of studies. In our study autologous whole blood injection lead to significant pain and functional improvement in chronic tennis elbow.

In a trial in 2010, Kazemi found that at 8 weeks post-injection that AWB appeared to be more efficacious in all outcomes (including pain and function) than steroid injection [P <0.001]. However, there was a high risk of bias in that study because of inadequate randomization method [10].

In 2012, Dojode et al. compared autologous blood injection to steroid injection in 60 patients with chronic tennis elbow. They found that the steroid group demonstrated better pain relief at 1 and 4 weeks follow-up. However, at 12 weeks and 6 months, there was significantly better pain reduction in the whole blood group than in the steroid group. Also, there was a greater recurrence rate in the steroid group compared to the AWB group (37% vs. 0%) [26].

However, recent reviews of clinical trials revealed limited evidence supporting the effectiveness of autologous blood injections for chronic tendinopathies. According to these reviews, even though refractory chronic tendinopathy might be responsive to AWBs and despite the proven efficacy of PRP on tissue regeneration in experimental studies, but the data available to date are limited by quality and size of study, as well as length of follow up and are currently insufficient to recommend this modality for routine clinical use [27-30].

In the present study, there was no significant difference in pain reduction and functional improvement between PRP and AWB injection in chronic tennis elbow in 12 month follow up. The effect of autologous whole blood in comparison with PRP has been investigated in some other studies; in a systematic review in 2009 by Rabago, four injection therapies for lateral epicondylosis: prolotherapy, polidocanol, whole blood and platelet-rich plasma, whole blood injections were assessed, They reported significant improvement in functional scores and in maximal grip strength compared with baseline in the intervention groups. They concluded that according to existing data for autologous whole blood and PRP injection, these therapies could be effective in treating tennis elbow, but as the authors concluded the results of this systematic review were limited by lack of large definitive clinical trials [31].

Creaney conducted a study of 150 people comparing whole blood to PRP for the treatment of lateral epicondylitis. The participants had all previously failed to respond to a more ‘conservative’ treatment like stretching and eccentric exercise. Using the criteria of an improvement of 25 points on the patient-related tennis elbow evaluation score (PRTEE), improvement was noticed in both groups and there was no significant difference in the success rate between either [32]. The results of our study were in agreement with the results of this study.

In a systematic review in 2012, conducted to determine the efficacy of autologous blood concentrates in decreasing pain and improving healing and function in patients with orthopaedic bone and soft-tissue injuries, the authors evaluated twenty three randomized trials and ten prospective cohort studies and concluded that there is uncertainty about the evidence to support the increasing clinical use of platelet-rich plasma and autologous blood concentrates as a treatment modality for orthopaedic bone and soft-tissue injuries including tennis elbow [33].

In a systematic review and network meta-analysis of randomized controlled trials by Krogh in 2013, the comparative effectiveness and safety of injection therapies in patients with lateral epicondylitis were assessed. Both autologous blood and platelet-rich plasma were also statistically superior to placebo in clinical trials [25].

Generally, there is moderate evidence from two fair quality (1+) RCTs that platelet-rich plasma is no more efficacious than autologous blood injections for the treatment of lateral epicondylitis [32,34].

The main factors which may cause controversy in the studies mentioned above regarding the efficacy of PRP or whole blood might arise from lack of standardization of study protocols, platelet-separation and injection techniques whether ultrasound guided or blind, and outcome measures.

Both PRP and whole blood therapies have been claimed to promote healing through the action of various growth factors on the affected tendon [35]. The mechanism of action is proposed to be a healing response in the damaged tendons triggered by the growth factors in the blood. These growth factors trigger stem-cell recruitment, increase local vascularity and produce an instructional biological microenvironment for local and migrating cell activities [36].

It is believed that platelet-rich plasma can augment or stimulate healing by turning on the same biological healing process that normally occur in the human body after musculoskeletal injury. However, not only platelet-rich plasma, but also platelet-poor plasma, stimulates cell proliferation and total collagen production [37,38]. Increased production of endogenous growth factors have been found in human tendons treated with PRP [3,12,21]. The above mechanism helps explain why PRP alone or whole blood application can have a lasting effect on the healing process [23-25].

## Conclusion

PRP and autologous whole blood injections are both effective methods to treat chronic lateral epicondylitis. The efficacy of PRP was similar to whole blood injection at 12 month follow up in relieving pain and improving function. It can be concluded from our study that there might be no need to platelets in higher concentration than whole blood to get therapeutic effects.

Because PRP and whole blood are autologous and are prepared at the point of care, they have an excellent safety profile.

The limitation of our study was the relatively small number of cases included and absence of a control group receiving no intervention assigned as the “wait and see” group. However, long term follow up of patients was a strong point for the present study.

We encourage more randomized clinical trials on this topic designed with low risk of bias to investigate the real efficacy of blood products compared to no treatment, also the best technique of PRP injection, number and time of injections and number of platelets. There is also need for standardisation of PRP preparation methods in all clinical trials.

## Competing interests

The authors declare that they have no competing interests.

## Authors’ contributions

AR was the leader of the project, MR was the consultant professor, HH participated in the manuscript preparation, RR recruited the patients and conducted follow up assessments, LS prepared the main manuscript and KR participated in PRP preparation and data analysis. All authors read and approved the final manuscript.

## Pre-publication history

The pre-publication history for this paper can be accessed here:

http://www.biomedcentral.com/2052-1847/6/12/prepub
